# Supplementary figures and images for: Antibody-induced internalisation of retroviral envelope glycoproteins is a signal initiation event
Source: PLoS Pathog. 2020 May 26;16(5):e1008605. doi: 10.1371/journal.ppat.1008605 (PMC7274472; doi:10.1371/journal.ppat.1008605)

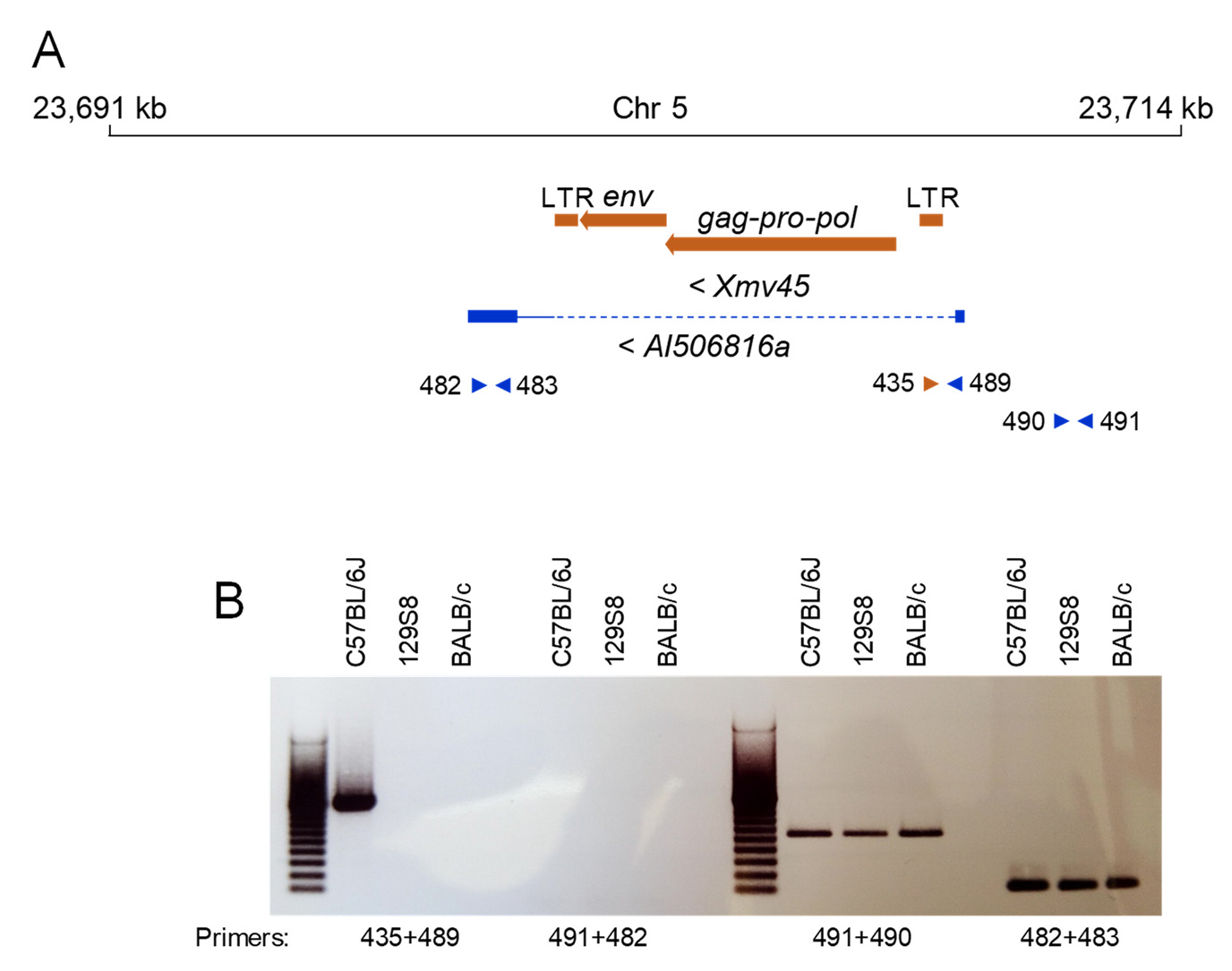

Supplement: S1 Fig — (A) Schematic representation of reconstructed locus prior to Xmv45 integration, referred to as AI506816a, depicting the position of PCR primers used. (B) PCR results from genomic DNA from the indicated inbred mouse strains. (TIF) [file ppat.1008605.s001.tif]

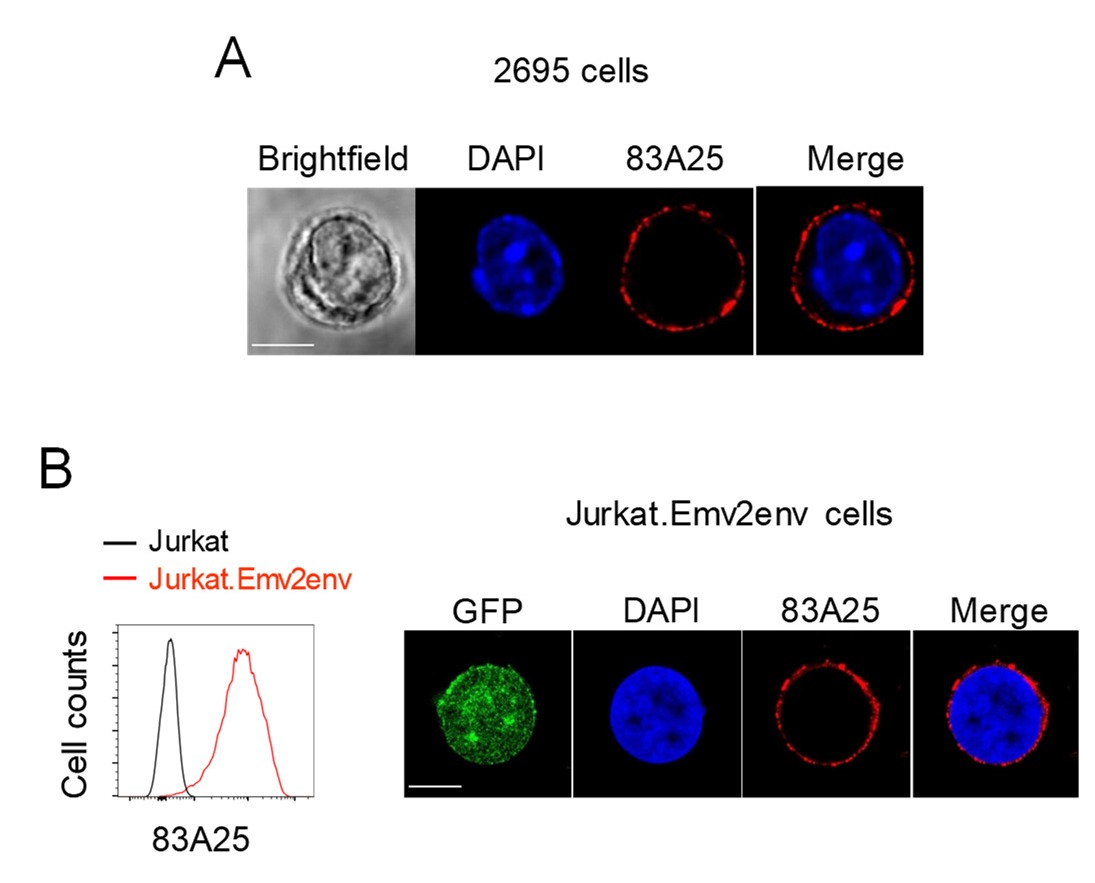

Supplement: S2 Fig — (A) Representative confocal images showing distribution of MLV envelope proteins from endogenous retroviruses in 2695 B cell lymphoma line. Cells were fixed, permeabilised and labelled with 83A25. Scale bar = 5 μm. (B) Flow cytometric analysis of Emv2 envelope expression on the surface of Jurkat.Emv2env cells (left) and representative confocal images showing distribution of Emv2 envelope in transduced Jurkat cells (right). Cells were fixed, permeabilised and labelled with 83A25. Scale bar = 5 μm. (TIF) [file ppat.1008605.s002.tif]

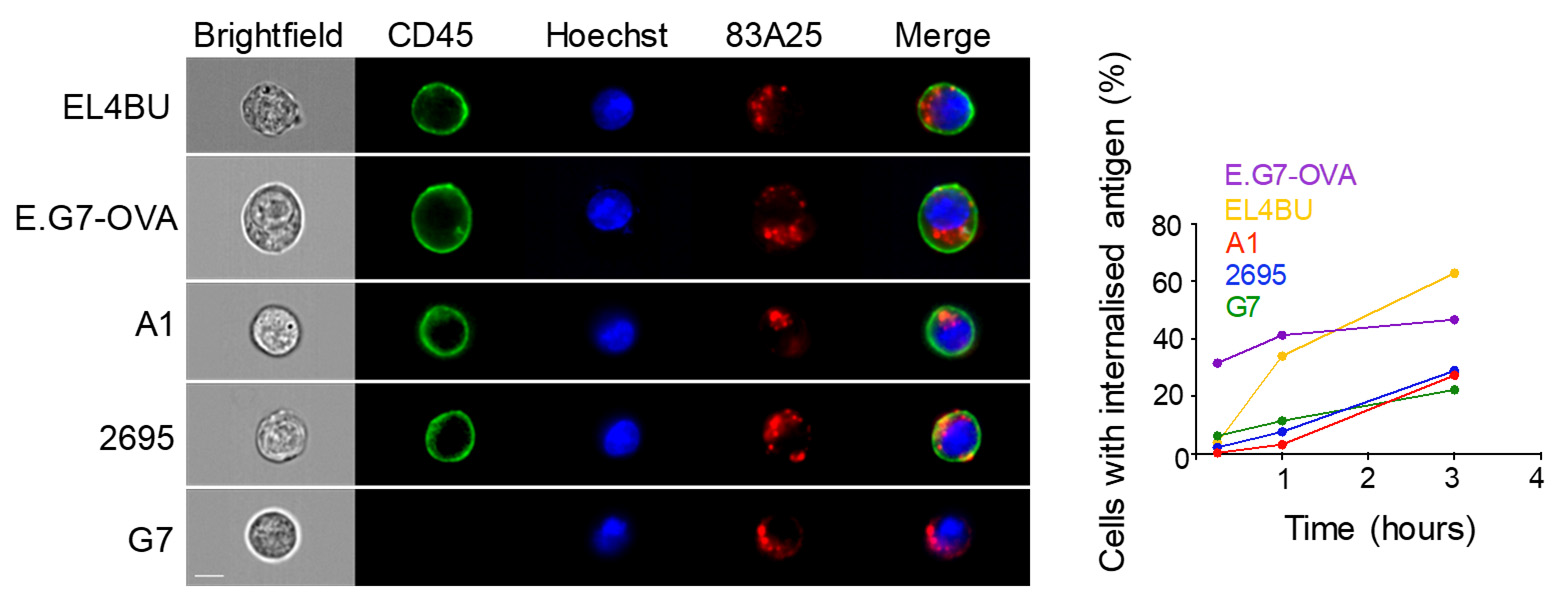

Supplement: S3 Fig — IS images of various tumour cell lines incubated with 83A25 for 3 hours and counterstained with anti-CD45 and Hoechst (left). Quantification of cells with internalised envelope-antibody complexes (right). A minimum of 5000 cells were analysed at each time point. Scale bar = 7 μm. (TIF) [file ppat.1008605.s003.tif]

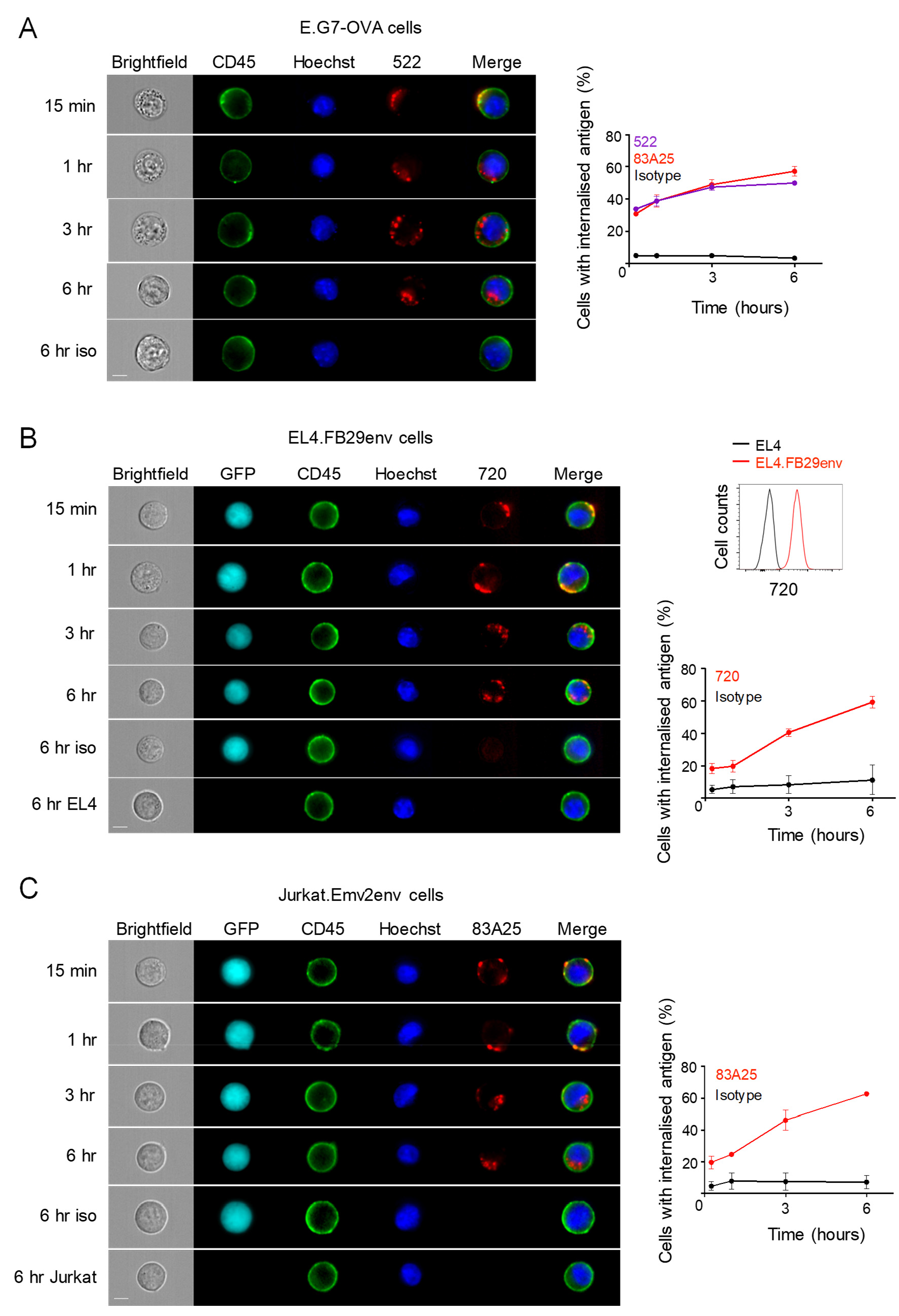

Supplement: S4 Fig — (A) Xenotropic envelope is internalised within three hours of incubation with the 522 antibody. IS images of E.G7-OVA cells incubated with 522 for specified periods of time and counterstained with anti-CD45 and Hoechst (left). Quantification of cells with internalised envelope/antibody complexes (right) from two independent experiments. A minimum of 10000 cells were analysed in each experiment at each time point. Scale bar = 7 μm. (B) F-MLV envelope is internalised within three hours of incubation with the 720 antibody. F-MLV FB29env-transduced EL4 cells were incubated with 720 for specified periods of time, counterstained with anti-CD45 and Hoechst and imaged by IS (left). Flow cytometric analysis of F-MLV FB29 envelope expression on the surface of EL4.FB29env cells (top right). Quantification of cells with internalised F-MLV FB29 envelope-antibody complexes (bottom right) from two independent experiments. A minimum of 10000 cells were analysed in each experiment at each time point. Scale bar = 7 μm. (C) Emv2 envelope is internalised within 3 hours of incubation with the 83A25 antibody. Emv2env-transduced Jurkat cells were incubated with 83A25 for specified periods of time, counterstained with anti-CD45 and Hoechst and imaged by IS (left). Quantification of cells with internalised Emv2 envelope-antibody complexes (right) from two independent experiments. A minimum of 10000 cells were analysed in each experiment at each time point. Scale bar = 7 μm. (TIF) [file ppat.1008605.s004.tif]

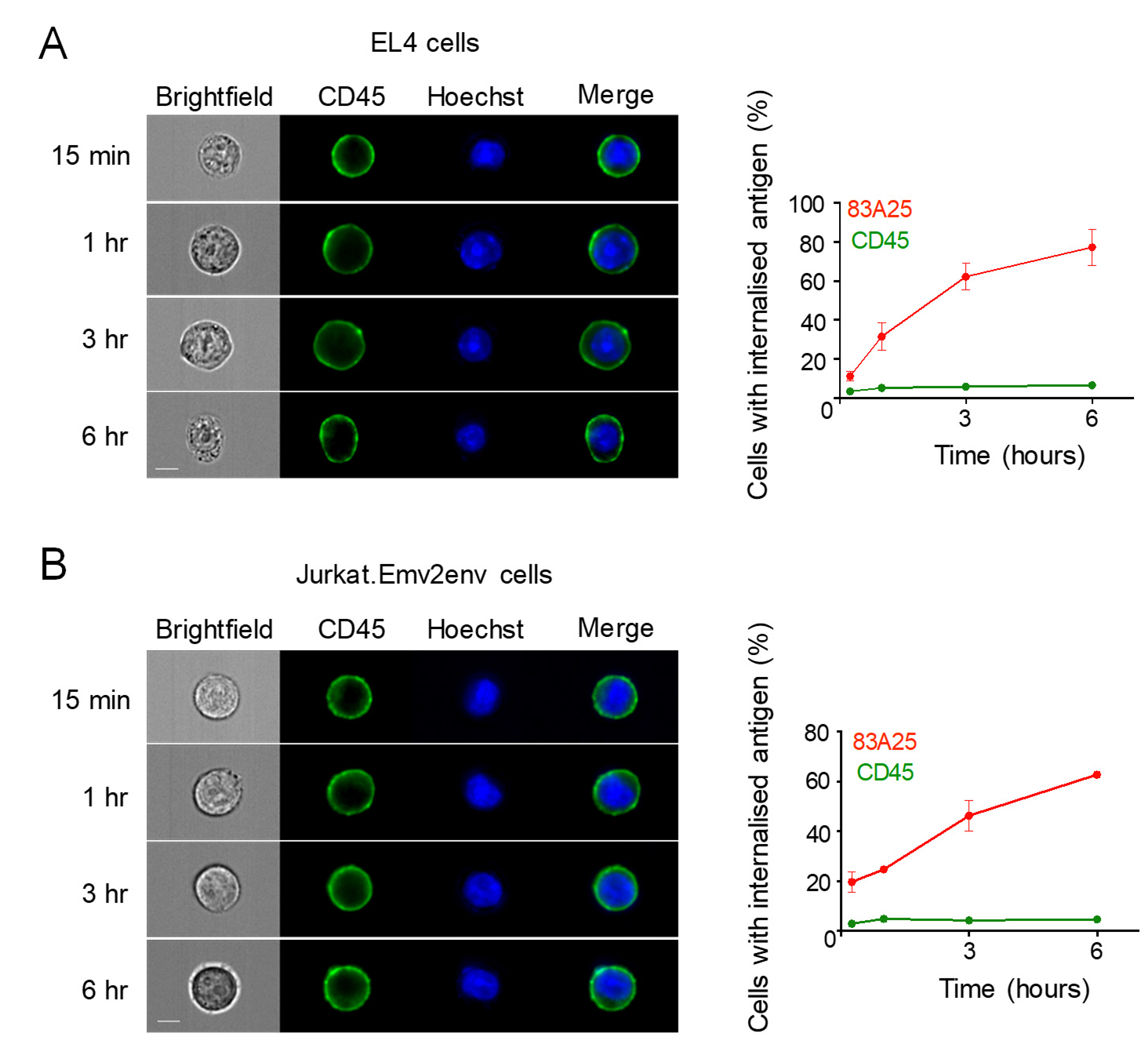

Supplement: S5 Fig — (A) IS images of EL4 cells incubated with anti-mouse CD45 for indicated periods of time and counterstained with Hoechst (left). Quantification of cells with internalised CD45-antibody complexes compared to envelope-antibody complexes (right). A minimum of 10000 cells were analysed at each time point. Scale bar = 7 μm. (B) IS images of Jurkat cells incubated with anti-human CD45 antibody for indicated periods of time and counterstained with Hoechst (left). Quantification of cells with internalised CD45-antibody complexes compared to envelope-antibody complexes (right). A minimum of 10000 cells were analysed at each time point. Scale bar = 7 μm. (TIF) [file ppat.1008605.s005.tif]

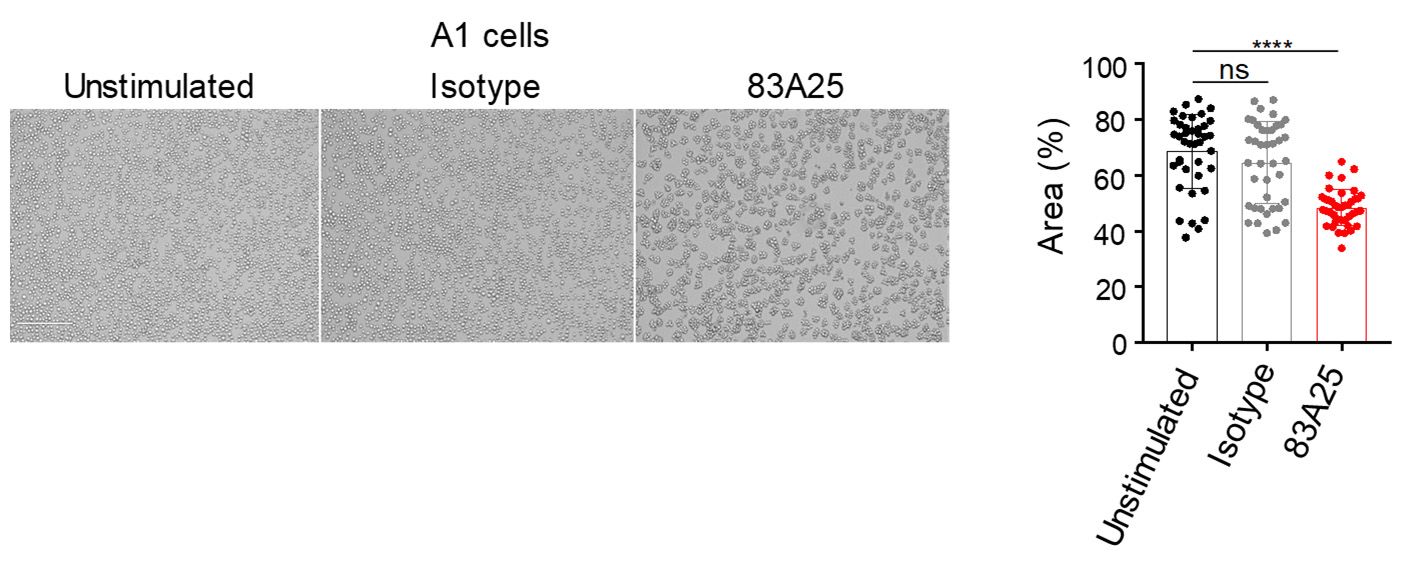

Supplement: S6 Fig — Light microscopy images of A1 cells incubated with indicated antibodies for 18 hours (left). Scale bar = 200 μm. Quantification of area occupied by the cells as a percentage of total per field of view (right). Pooled data from three independent experiments with at least ten fields of view per experiment. (TIF) [file ppat.1008605.s006.tif]

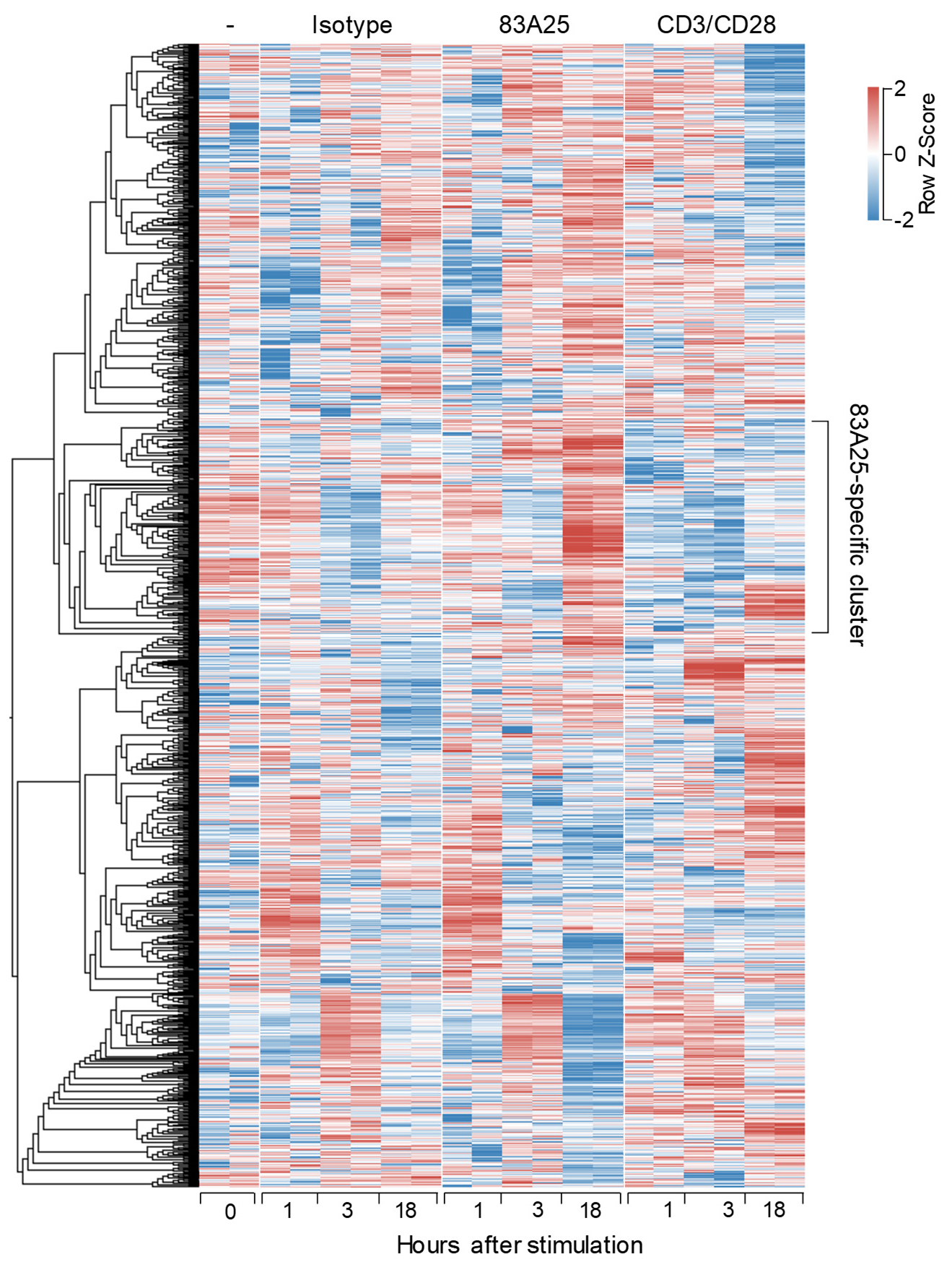

Supplement: S7 Fig — Changes in gene expression in EL4 cells over time following incubation with 83A25, isotype control antibody or CD3 and CD28 Dynabeads. Each column is an independent replicate. (TIF) [file ppat.1008605.s007.tif]

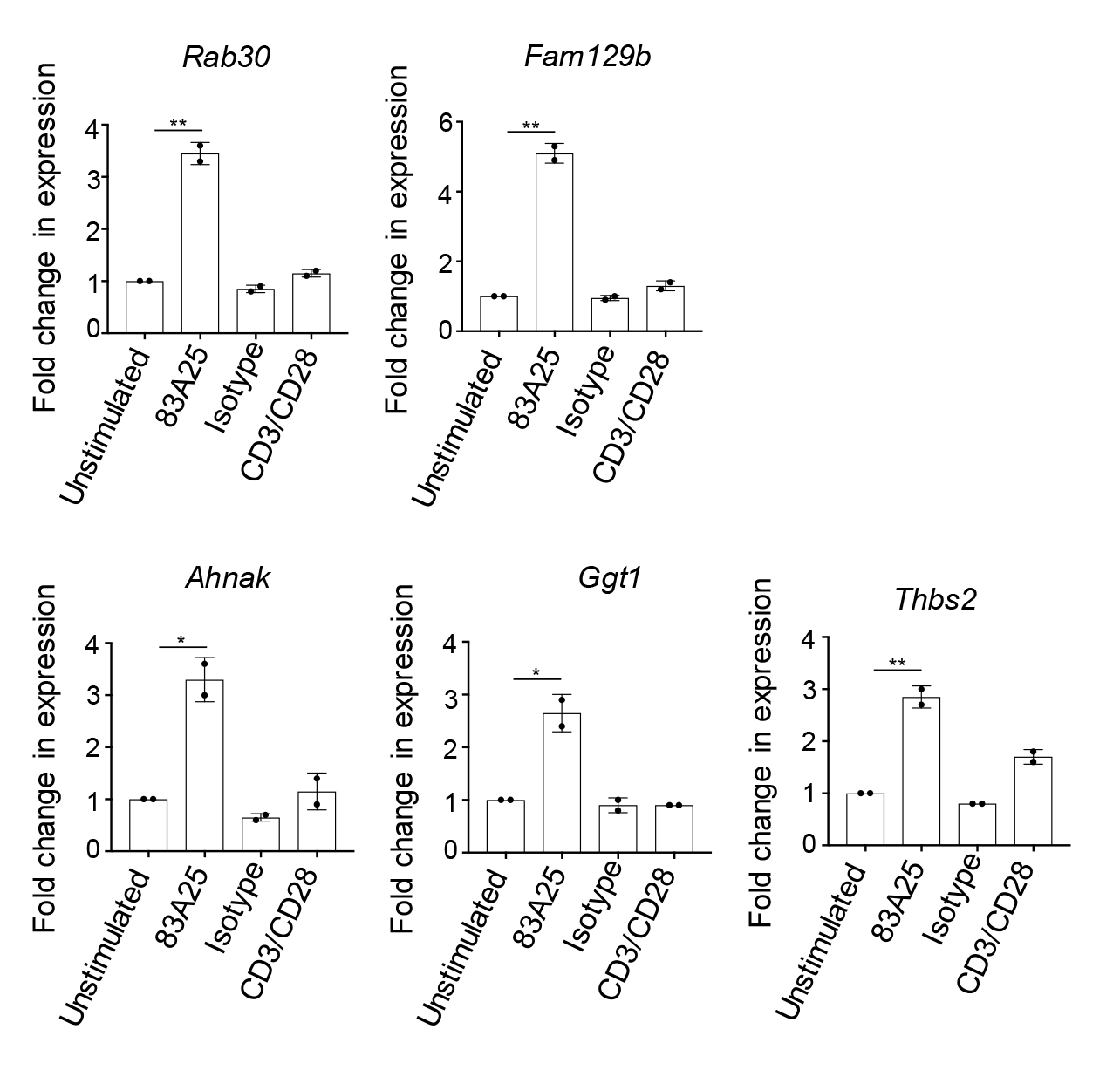

Supplement: S8 Fig — Expression of Rab30, Fam129b, Ahnak, Ggt1and Thbs2 genes assessed by qRT-PCR in EL4 cells stimulated with 83A25 for 18 hours. (TIF) [file ppat.1008605.s008.tif]

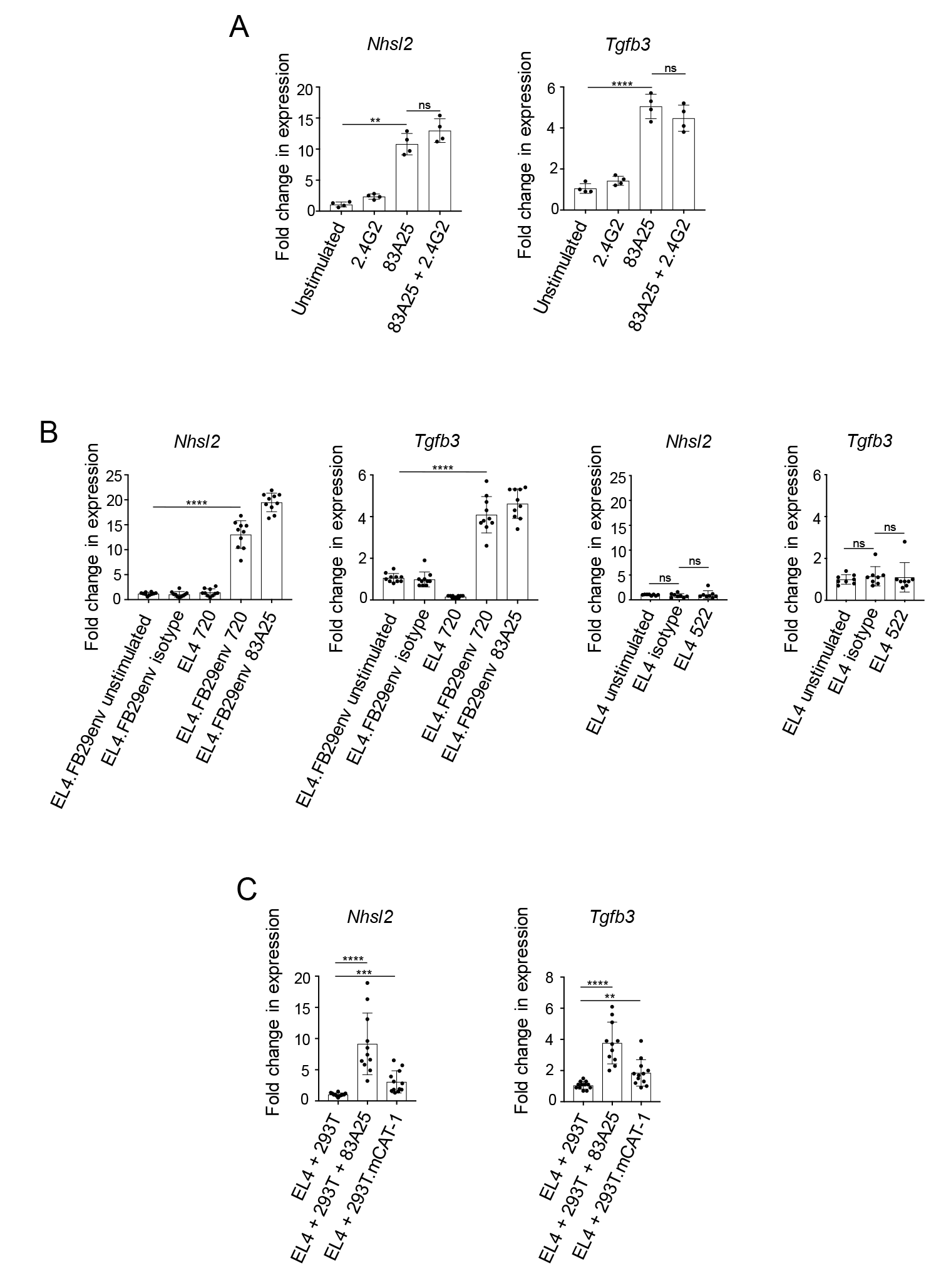

Supplement: S9 Fig — (A) Expression of Nhsl2 and Tgfb3 genes assessed by qRT-PCR in EL4 cells stimulated with 83A25 for 18 hours in the presence of 2.4G2 FcR blocking antibody. (B) Expression of Nhsl2 and Tgfb3 genes assessed by qRT-PCR in EL4.FB29env cells stimulated with the 720 antibody and in EL4 cells stimulated with the 522 antibody for 18 hours. Pooled data from two (522) and three (720) independent experiments. (C) Expression of Nhsl2 and Tgfb3 genes assessed by qRT-PCR in EL4 cells co-cultured with 293T or 293T.mCAT-1 cells for 18 hours. Pooled data from three independent experiments. (TIF) [file ppat.1008605.s009.tif]

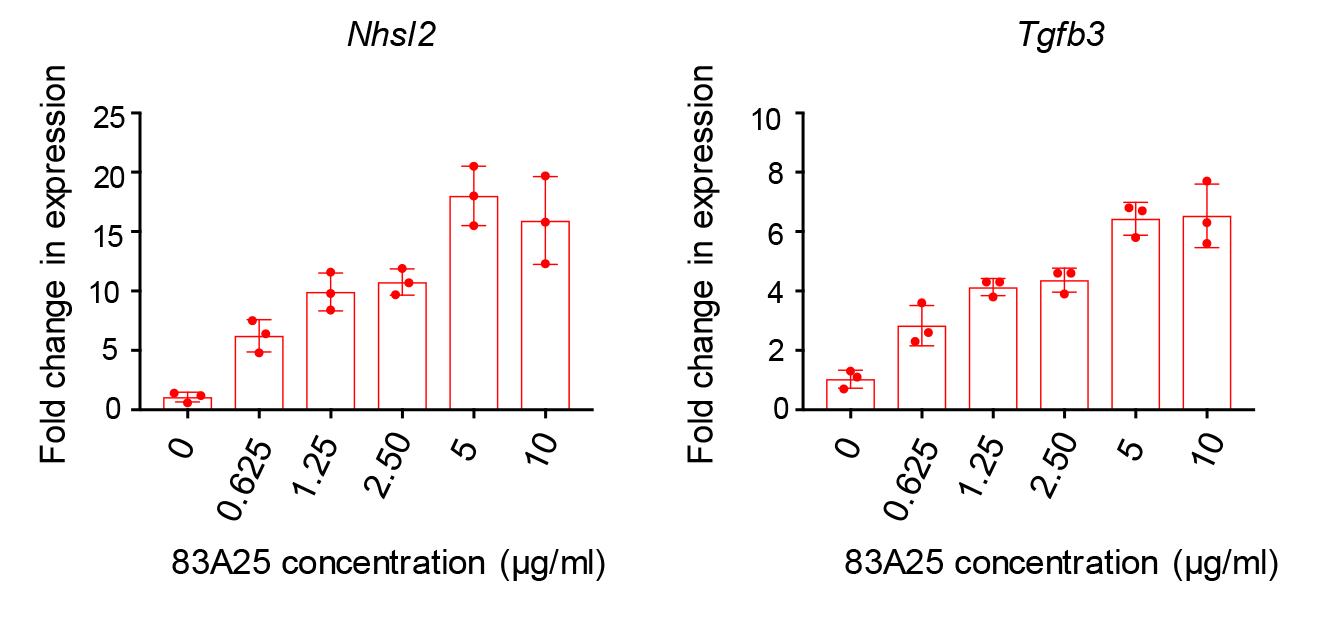

Supplement: S10 Fig — EL4 cells were stimulated with various concentrations of 83A25 for 18 hours and levels of Nhsl2 and Tgfb3 gene expression were assessed by qRT-PCR. (TIF) [file ppat.1008605.s010.tif]

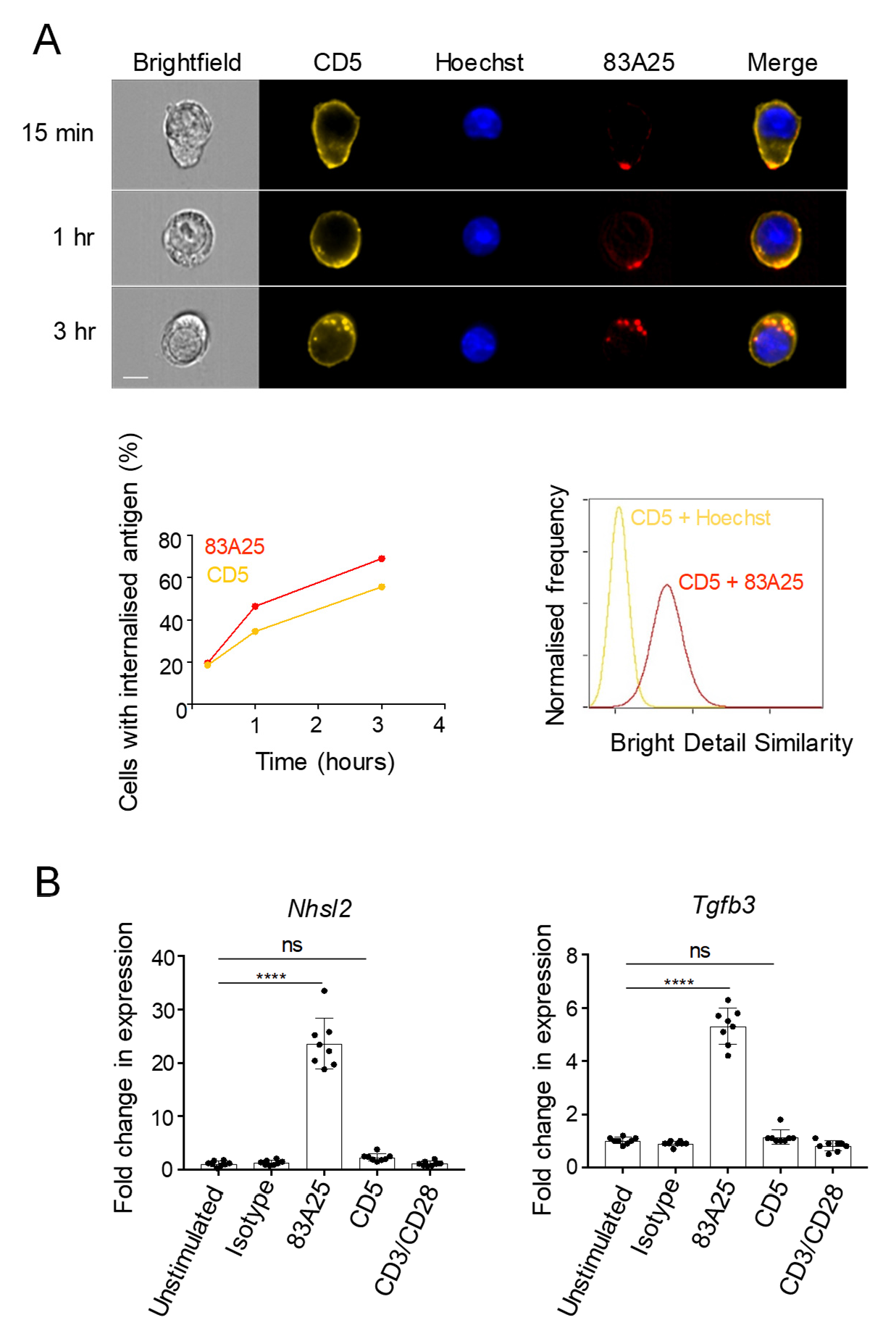

Supplement: S11 Fig — (A) CD5 is internalised into the same vesicles as 83A25-envelope complexes. IS images of EL4 cells co-incubated with 83A25 and anti-CD5 for specified periods of time and stained with Hoechst (top panel). Scale bar = 7 μm. Quantification of cells with internalised envelope-antibody complexes (bottom left). A minimum of 5000 cells were analysed at each time point. Co-localisation of 83A25 with CD5 was quantified using the Bright Detail Similarity feature in IDEAS and compared to Hoechst, a non-colocalising probe (bottom right). (B) Expression of Nhsl2 and Tgfb3 genes assessed by qRT-PCR in EL4 cells stimulated with anti-CD5 for 18 hours. Pooled data from two independent experiments. (TIF) [file ppat.1008605.s011.tif]

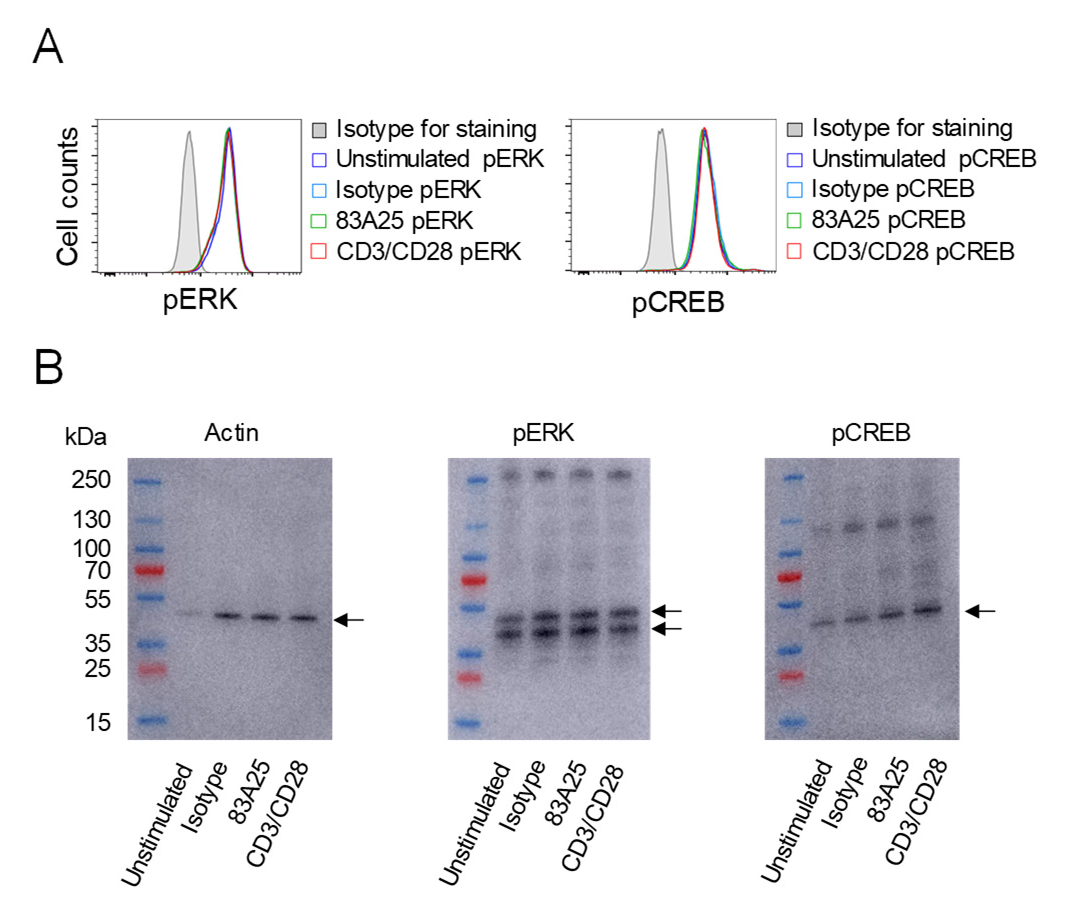

Supplement: S12 Fig — (A) Flow cytometric analysis of intracellular phospho-ERK (pERK) and phospho-CREB (pCREB) in resting EL4 cells and following stimulation with the indicated antibodies for 20 minutes. Grey-filled histograms represent the isotype control for the staining. Data representative of three independent experiments. (B) Western blot analysis of pERK and pCREB in resting EL4 cells and following stimulation with the indicated antibodies for 20 minutes. Data representative of one experiment. (TIF) [file ppat.1008605.s012.tif]

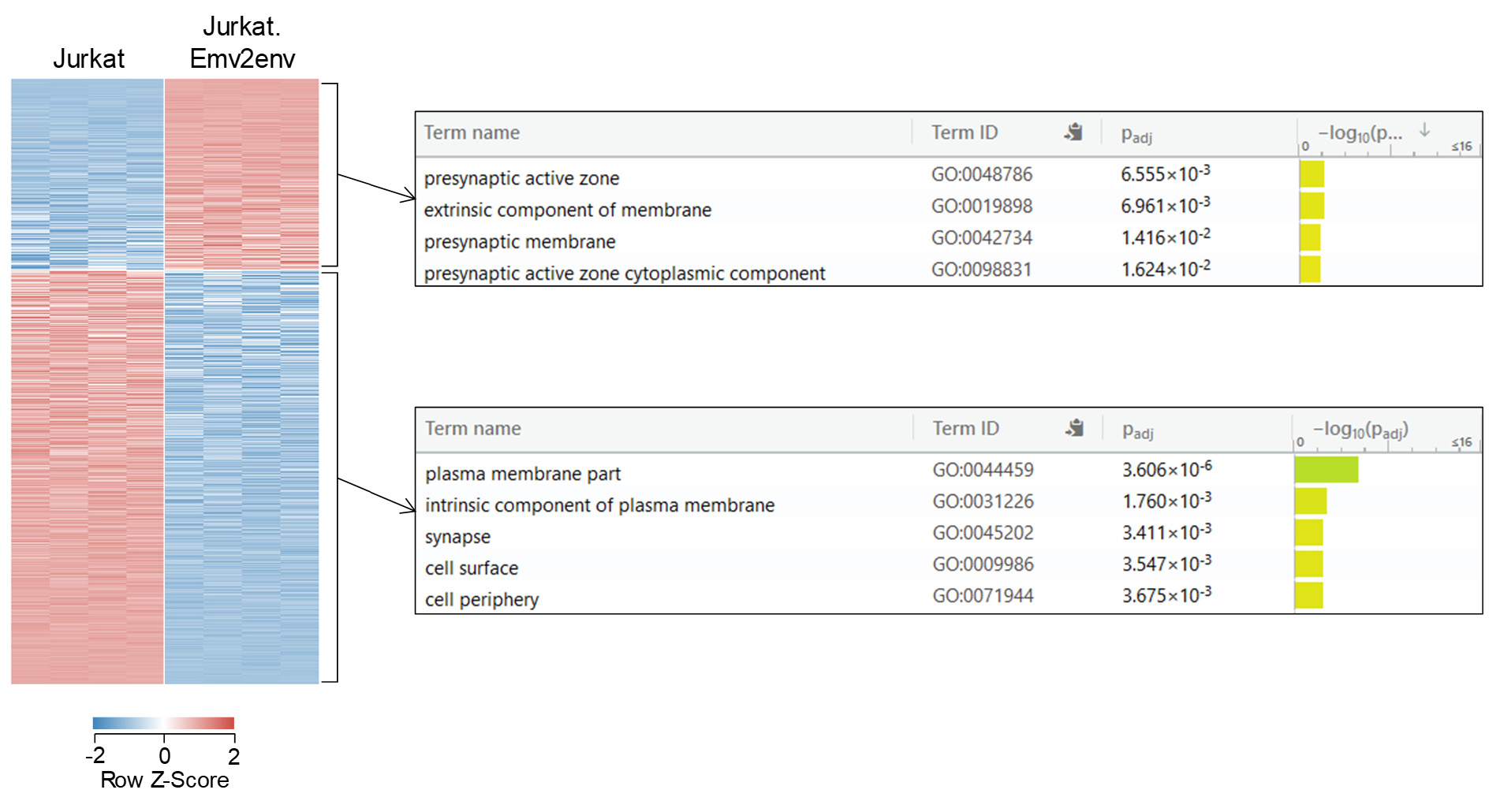

Supplement: S13 Fig — Heatmap of differentially expressed genes (≥2-fold, q≤0.05) between Jurkat and Jurkat.Emv2env cells (left) and pathway analysis of these genes, according to g:Profiler (https://biit.cs.ut.ee/gprofiler). (TIF) [file ppat.1008605.s013.tif]

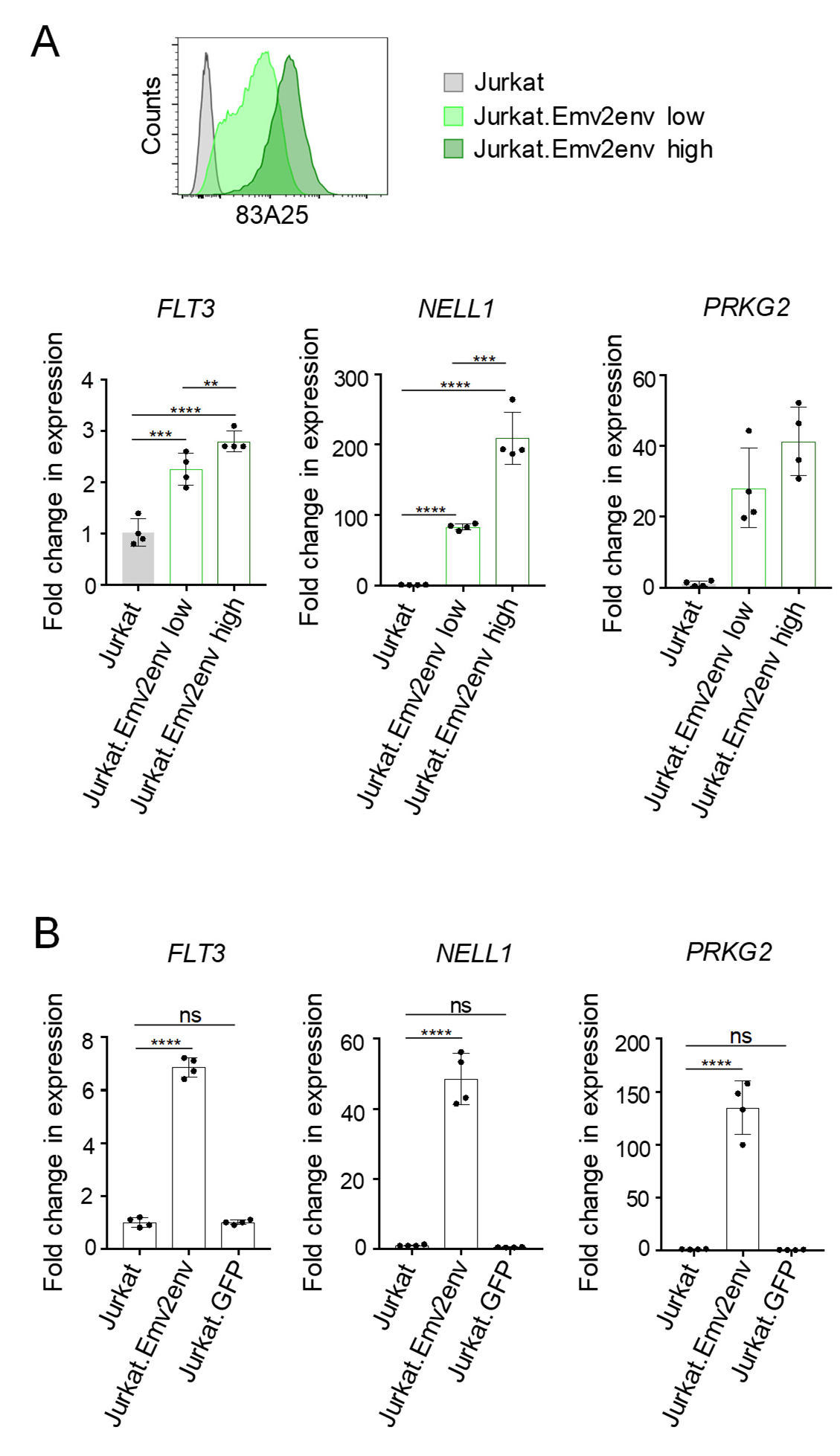

Supplement: S14 Fig — (A) FLT3, NELL1 and PRKG2 gene expression correlates with Emv2 envelope expression levels on the cell surface. Jurkat.Emv2env cells were sorted for Emv2 envelope low or high (top) and assessed for expression of FLT3, NELL1 and PRKG2 genes by qRT-PCR (bottom). (B) Verification of differentially expressed genes by qRT-PCR analysis. Expression of FLT3, NELL1 and PRKG2 genes in Jurkat.Emv2env and Jurkat.GFP cells assessed by qRT-PCR. (TIF) [file ppat.1008605.s014.tif]

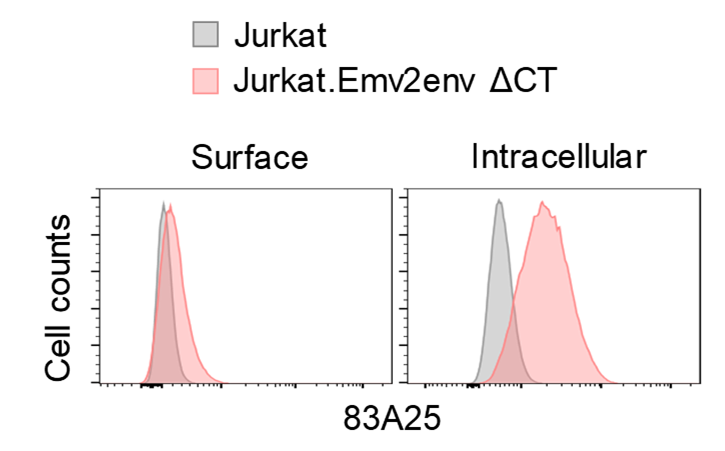

Supplement: S15 Fig — Flow cytometric analysis of Jurkat.Emv2env ΔCT cells for surface (left) and intracellular (right) expression of Emv2 envelope. (TIF) [file ppat.1008605.s015.tif]
