# Supplementary material for: Antibody-induced internalisation of retroviral envelope glycoproteins is a signal initiation event
Source: PLoS Pathog. 2020 May 26;16(5):e1008605. doi: 10.1371/journal.ppat.1008605 (PMC7274472; doi:10.1371/journal.ppat.1008605)
Supplement: S1 Table — (PDF) [file ppat.1008605.s016.pdf]

S1_Table: Sequence of PCR primers used in this study

Primer name	Primer sequence
435	CCGAAAGCAAAAATTCAGATGGAG
482	GGTTGGCACTGTAGGCTTTG
483	AGACTTAGGGAAGGAGCGGA
489	CCCAGGCATGAGCAGAGTAA
490	ATGGTTTAGAGGTTGCCTTGCT
491	TTGGAGATTTTCACGGCGCT

Gene name	Forward primer sequence	Reverse primer sequence
mNhsl2	AGCTGCTGAAGACCACTAAC	TTAGGTACTGGGGTTGTCAG
mTgfb3	TCGGAGTCTGAGTACTATGC	TCATCCGGTCGAAGTATCTG
mAbhd12b	GGACGCTAGGGACTGTGATG	AAGTTTCTGTACTCGGCAGC
mPmaip1	CAGAGCTACCACCTGAGTTC	GAGCACACTCGTCCTTCAAG
mRab30	CGTGCCTAGTCCGAAGATTC	GTAACTTTGAGTGATGGAGCG
mFam129b	AGACGTACTGTCCACACACC	CTCGATCTCATGGCGCATGC
mAhnak	GACCAGATTGTGGGTGCCAC	ATGCGCTGGTAGTCCTCATC
mGgt1	CAGAGATTGGACGGGATATC	CCTTGGAGTTGTTGAACATGG
mThbs2	GTGCATCTCGAGAGAGTCAC	GTGCGTACACACCTCCTGTG
mIl2	GTGCTCCTTGTCAACAGCG	GGGGAGTTTCAGGTTCCTGTA
hEGR1	CCAGCCAAACCACTCGACTG	CTAGGCCACTGACCAAGCTG
hFLT3	CACTCGAGGAGGGCAACTAC	GCTTCTGCGAGCACTTGAGG
hNELL1	TCAGATGGATATCGTCACCG	ACCGAATCTCATCCCTCAGG
hPRKG2	AGCTCCAGAACAAGTGCATC	TGTAACTCCCTTGCTGATAG
FB29 env	GCCTAGACCTGTTGTTCCTAAAAGAG	TGGCCATACTATCTCTTACTAG
Emv2 env	AGGCTGTTCCAGAGATTGTG	TTCTGGACCACCACACGAC
