# Supplementary material for: Antibody-induced internalisation of retroviral envelope glycoproteins is a signal initiation event
Source: PLoS Pathog. 2020 May 26;16(5):e1008605. doi: 10.1371/journal.ppat.1008605 (PMC7274472; doi:10.1371/journal.ppat.1008605)
Supplement: Attachment — Submitted filename: point-by-point reply.pdf [file ppat.1008605.s017.pdf]

We thank all reviewer for the astute and constructive criticism. Below is a point-by-point list of the changes we have made.

Major Issues: Key Experiments Required for Acceptance

Reviewer #1:

Comment 1- Previous studies have shown that antibody-mediated Env internalisation depends on their capacity to crosslink. Fab fragments appear to be unable to induce Env internalisation. Is this also true for MLV envelope glycoproteins? Does 83A25 Fab fragment induce internalisation?

Reply: We have attempted to test if Fab fragments of the 83A25 antibody induces internalisation. 83A25 Fab were prepared with the Pierce F(ab')2 Preparation Kit and conjugated to Alexa Fluor 647 as the intact antibody. However, 83A25 Fab failed to bind MLV envelope on EL4 cells with sufficient affinity to allow us to detect the signal and follow its internalisation. This might be expected given the reduced overall binding affinity of Fab, compared with intact antibodies.

Comment 2- Similarly, is antibody-mediated crosslinking required to signal? This could be easily tested in several ways, for example by measuring by qRT-PCR the upregulation of Nhsl2 RNA (Figure 3F) induced by 83A25 compared to its Fab fragment.

Reply: Given the inability of 83A25 Fab to bind MLV envelope with sufficient affinity, these fragments could not be used to determine whether cross-linking is required. Instead, two pieces of information presented in the manuscript argue that cross-linking is not required. Firstly, blocking of Fc receptors with the 2.4G2 antibody did not block the induction of Nhsl2 and Tgfb3 transcription by 83A25 (Fig. S9A). Secondly, plate-bound 83A25, which represents the cross-linked form, did not induce Nhsl2 or Tgfb3 transcription (Fig. 4A).

Comment 3- Is the transcriptional activation and signalling unique to 83A25 or also observed with 522 and 720? (these antibodies also induce Env internalisation).

Reply: We have also tested the 720 and 522 antibodies. The results for both are now shown in Figure S9B. The 720 antibody induces transcription of Nhsl2 and Tgfb3 in EL4 cells expressing its target (the FB29 envelope), at levels comparable with 83A25 stimulation of EL4 cells. In contrast, the 522 antibody did not stimulate transcription of either gene. Thus, this property is not unique to 83A25, but it is not universal either. It may be that only antibodies that react with ecotropic MLV envelopes trigger signalling. Alternatively, it could be this this requires IgG classes of antibody, and the 522, which is the only IgM in our set cannot stimulate. These possibilities are now discussed.

Reviewer #2:

Comment 1) Most of presented data are obtained by using cultured cell lines and transfectants. While it is clear that the ligation of cell surface envelope molecule either with an antibody or with mCAT-1 receptor induces envelope protein internalization and cytoplasmic YXXphai motif is required for this process, and the above envelope ligation results in the induction of a battery of genes in EL4 and transfected Jurkut cells that are different from those induced by CD3/CD28 stimulation, it is unclear if this unique signaling is associated with any particular type of cell activation in primary T and B lymphocytes or other cells of the immune system.

Reply: We agree with the Reviewer that our study is limited to cell lines and lacking data with primary lymphocytes. We have treated primary cells with 83A25 to examine cellular activation, but these experiments were unsuccessful. This is because primary lymphocytes do not survive in culture for the period of time required to see the effects of 83A25 unless they are stimulated also through the antigen receptor (CD3) or with growth factors (IL-2 or IL-7), which masks any effect of 83A25. Indeed, primary T and B cells will not survive an overnight culture, and although the addition of 83A25 is not sufficient to keep them alive, we cannot conclude on any signalling event in primary cells.

Comment 2) In this regard, while the authors describe that MLV envelope glycoprotein expression is linked with immune activation and antibody responses elicited against endogenous retroviral envelope proteins in the Abstract and Author Summary, they have failed to cite and discuss about important previous reports that are highly relevant to the relationship between immune activation and antibody production against endogenous retroviral envelope glycoproteins. John Portis and his colleagues isolated a large number of monoclonal antibodies reactive with endogenous xenotropic and polytropic viral envelope glycoproteins by inducing graft-versus-host reaction (Portis, J. L., F. J. McAtee, M. W. Cloyd. Virology 118:181-190, 1982; Portis, J. L. and F. J. McAtee. Virology 126:96-105, 1983). Generation of a large number of hybridomas reacting to endogenous retroviral rather than host antigens was unexpected, and he later discussed the possibility of antigen-driven activation of autoreactive lymphocytes (Portis, J. L. Tohoku J. Exp. Med. 173:83-89, 1994). The authors should cite these papers and discuss the possible relationships between their observation and the induction of antiretroviral antibodies upon immune activation.

Reply: We thank the Reviewer for drawing our attention to these reports. They have now been cited and discussed appropriately.

Comment 3) In Figure 1, the authors first examined the expression of endogenous retroviruses in unstimulated and stimulated CD4+ T cells and then abruptly switch to LPS stimulation of B cells. While the genetic analyses on the mechanisms of LPS responsiveness are very important, is there any rationale to start with CD4+ T cells, switch to LPS stimulation of B cells, and come back to EL-4 T cell line?

Reply: We apologise for not making this clearer in the original submission. The high expression of Xmv45 in B cells had already been demonstrated in our earlier publication (Attig et al., 2017), which is why it was not included in this one. Also, the induction of endogenous MLVs by LPS stimulation of B cells has been extensively described. Instead, here we focus on Xmv45 transcription in T cells, where it is less understood. We now show that in both B cells and T cells, Xmv45 transcription is driven by the AI506816 promoter. We reverted to using B cells for Xmv45 induction, as this provirus is not inducible by CD3/CD28 stimulation of primary T cells (Fig. 1A). We have now added data to

show that this provirus is not inducible by CD3/CD28 stimulation of EL4 cells either (Fig. 1E). Thus, the inducibility of Xmv45 could only be studied in B cells.

Comment 4) In this regard, the expression of endogenous xenotropic viral envelope gene(s) and the production of "autoantibodies" reactive with the envelope glycoprotein have long been associated with the pathogenesis in lupus-prone mice as the authors briefly mention, and a lack of association of antinuclear antibodies with glomerulonephritis or vasculitis (Christensen, S. R. et al. Immunity 25:417-428, 2006) and direct pathogenicity of autoantibodies reactive with "xenotropic" viral gp70 have been reported (Tabata, N. et al. J. Virol. 74:4116-4126, 2000). Of particular importance, retroviral gp70-containing circulating immune complexes are associated with the development of glomerulonephritis in murine models of SLE and the source of serum gp70 is the liver where gp70 is expressed as an acute phase reactant (Shigemoto, K. et al. Mol. Immunol. 29:573-582, 1992). Thus, there must be some analyses and discussion on the possible relationship between the LPS-responsive Xmv45 and the gene encoding the serum gp70 that is expressed as acute phase reactant.

Reply: We thank the Reviewer for raising this issue, which has now been discussed. The induction of serum gp70 in certain strains of mice is another good example of integration of endogenous MLVs into the immune response. The production of gp70 is genetically controlled and mouse strain specific, depending both on the complement of endogenous MLVs and the allele at the Gv1 locus, which has been recently cloned by Treger et al. Xmv45 is one of the few endogenous MLV loci that are not controlled by Gv1 and is unlikely to be a major contributor to serum gp70. Nevertheless, there are very interesting parallels between the inducibility of Xmv45 and serum gp70 that are now discussed.

Reviewer #3:

Comment 1. In Figure 1, the authors show that Xmv45 is highly expressed in CD4 T cells but describe a chimeric transcript of Xmv45-AI506816a that is induced in LPS-activated B cells. What is the relevance of this finding? Is this chimeric transcript also expressed in activated T cells? Based on RNA sequencing, primary CD4+ T cells have decreased expression of Xmv45 upon CD3/CD28 stimulation (Figure 1a). How does this translate to surface expression of envelope in all of these cell types? Does CD3/CD28 stimulation also induce Xmv45 expression in EL4 cells, which are used in the majority of the experiments?

Reply: We have now clarified that all lymphocytes express the chimeric AI506816-Xmv45 transcript and not the full provirus. We have modified Fig. 1B to include the RNA-seq read mapping for T cells. The promoter of the AI506816 transcript, and not the proviral LTR, drives constitutive transcription of the env gene in T cells, which is not affected by CD3/CD28 stimulation in primary T cells, and we now show similar data for EL4 cells (Fig. 1E).

Comment 2. The authors claim that MLV envelope induction upon LPS stimulation is absent in 129S8 and BALB/c (Figure 1D) but the data lack unstimulated controls for each strain, thus the induction cannot be assessed.

Reply: We have now added the unstimulated controls for each strain. These are in addition to LPS stimulated, but unstained samples, as LPS stimulated B cell blasts exhibit higher auto-fluorescence than resting B cells. The combination of these two controls in the new Fig. 1D shows that envelope expression after LPS stimulation of 129S8 and BALB/c B cells is reduced and absent, respectively.

Comment 3. As demonstrated in Figure 2B, CD3 and CD19 are similarly endocytosed in primary lymphocytes upon 83A25 engagement (Figure 2B). These data suggest that the downstream signaling effects of 83A25-dependent envelope internalization described in EL4 cells (Figure 3) are potentially confounded by signaling through the internalized CD3. The authors should determine whether downstream consequences of envelope internalization (clustering, degranulation, transcriptional changes) are independent of CD3 internalization in EL4 cells.

Reply: We apologise for the lack of clarity. CD3 stimulation was used only to generate activated T cells. This stimulus was not present when activated T cells were subsequently incubated with 83A25. These cells were stained again with CD3 and CD45 antibodies at the end of treatment with 83A25 in preparation for imaging. Similarly, no CD3 antibody was present during EL4 cell treatment with the 83A25 and were only stained with the additional antibodies after fixation at the end of the treatment period.

Comment 4. The authors show that CD107a expression by EL4 cells stimulated with 83A25 is comparable to engagement of the envelope protein by the cognate receptor mCAT1. The authors should show whether mCAT1-dependent CD107a expression requires the same internalization process as 83A25 stimulation, and if not, explain the mechanism of internalization versus receptor engagement on downstream signaling.

Reply: The Reviewer raises the interesting point of how engagement of the cognate receptor mCAT1 by the eMLV envelope could lead to the same outcome as ligation with an antibody if only the latter can be internalised. We have now cited recent work by Izumida et al., who recently demonstrated that upon interaction between eMLV envelope-expressing cells and mCAT1-expressing cells, surprisingly the eMLV envelope-expressing cells internalise fragments of the target mCAT1-expressing cells. This suggests a general ability of eMLV envelope to internalise its ligands, whether these are soluble antibodies or transmembrane proteins.

Comment 5. The authors show that Nhsl2 and Tgfb3 induction upon 83A25 stimulation of EL4 cells is dependent on protein kinase A, Abl kinase, and Src kinase using chemical inhibitors. These results would be strengthened by demonstration that these pathways are indeed activated upon 83A25 engagement.

Reply: We have now investigated the pathways leading to pERK and pCREB activation, both by Western blotting and by flow cytometric staining with pERK and pCREB specific antibodies. As would be expected, these pathways were indeed constituvely activated in EL4 cells. These results are shown in the new Fig. S12. Their constitutive activation does not allow us to demonstrate further induction by 83A25 (or indeed by CD3/CD28) stimulation, but the results do argue that signalling via these pathways is both present and necessary.

Comment 6. Based on experiments in mouse cells, the authors conclude that antibody-mediated internalization of the envelope protein from the plasma membrane to the endocytic compartment results in activation of cellular signaling. However, in human Jurkat cells, the authors show that transcriptional changes requires the sorting motif within the cytoplasmic tail, even though internalization of the envelope protein is only partially affected by this motif. These data seem to decouple internalization of envelope from transcriptional changes and contradict their main conclusion.

Reply: With respect, our interpretation of these findings is different from the Reviewer's. We acknowledge that over-expression of retroviral envelopes in cells can create unphysiological situations that bypass normal controls. Nevertheless, our findings with over expression of Emv2 env in Jurkat cells support rather than contradict our main conclusion for the following reasons: Mutations in the sorting motif within the cytoplasmic tail do in fact reduce, but not abolish the internalisation of Emv2 envelope. Although the rate of internalisation following 83A25 ligation is not affected by sorting motif mutations, the total amount of internalised complex is reduced by half (Fig. 5E). Importantly, although internalisation is not completely prevented by sorting motif mutations, the transcriptional activation of the target genes (FLT3, NELL1 and PRKG2) is abolished. This means that the residual internalisation is not sufficient for signalling and additional function of the sorting motif are important. A contradiction of our main conclusion would be the opposite outcome of this experiment. If internalisation were abolished by sorting motif mutations, but signalling were not, then we would agree with the Reviewer that internalisation is not necessary. Instead, we find that internalisation is only partly inhibited, but signalling is blocked, which only means that internalisation (at least to the level seen with the mutants) in not sufficient, not that it is not necessary.

Minor Issues: Editorial and Data Presentation Modifications

Reviewer #1:

Comment: The authors suggest (in the abstract) that retroviral Env signalling could be a target for intervention. It is unclear how this would be achieved. For example, anti-Env Abs appear to induce signalling. Which intervention do the authors have in mind? Small molecules? Genetic approaches? Consider discussing further this intriguing idea.

Reply: We do not yet understand the physiological or pathological relevance of envelope signalling upon antibody ligation, so we can only speculate as to whether this pathway should in enhanced or inhibited. However, in practical terms, the pathway can be induced with stimulating antibodies or inhibited with downregulation of envelope expression. The latter can be achieved by epigenetic means, and although Xmv45 envelope expression is driven by a host promoter, the provirus should still be subject to epigenetic repression (e.g. by Cas9 repression systems). This is now discussed in the last paragraph of the Discussion.

Comment: Antibody-induced Env internalisation is not new and was previously-described for several viruses. I was surprised to see that this was only mentioned in the discussion; completely absent from the introduction section of the manuscript.

Reply: We have mentioned prior reports on internalisation now also in the introduction, but kept this brief to avoid a very lengthy introduction.

Comment: A better description on how the % of internalisation was calculated will facilitate the understanding of all the internalisation graphs presented in the manuscript.

Reply: We should clarify that the presented plots show simply the percentage of cells that have internalised antigen, not the degree of internalisation. Cells that had internalised antigen were identified using the IDEAS 6.2 internalisation wizard and CD45 surface stain as a marker of cellular membrane (except the CD45-negative G7 cell line where brightfield image was used as the cell image). However, the plots show only the frequency of such cells within the population, which was calculated by flow cytometric gating. This has been clarified in the legends.

Comment: On page 10 (result section describing Figure 3E), the authors should explain the rationale behind focusing only on Nhsl2, Tgfb3, Abhd12b and Pmaip1 out of all the genes that are upregulated upon 83A35 stimulation. What do these genes transcribe for and what is their relevance in signalling pathways as previously described in the literature? Similarly, the rationale for studying only Nhsl2 and Tgfb3 transcription in their antibody-immobilization assay and signalling inhibition assays in Figure 4.

Reply: We have now explained in the text that we focused on transcription of Nhsl2 and Tgfb3 because they were two of the most responsive genes specifically to 83A25 stimulation and provided a sensitive readout. This choice was not based on particular pathways or function for these genes.

Reviewer #2:

Comment 1) In Figure 1 legend, an "immune response gene" means a host gene that regulate immune responsiveness like those in the major histocompatibility complex. The authors here are referring to a gene that is responsive to immunologic stimulations, which is not described as immune response gene.

Reply: We thank the Reviewer for the correction, which has now been made.

Comment 2) In Figures 3, 4 and 5, statistical comparisons are made between multiple groups but the Materials and Methods describes only the use of one-way ANOVA and it is unclear if any post-hoc tests were performed for multiple comparisons. Further, while multiple asterisks are shown probably to indicate different levels of statistical significance, no explanations are provided in corresponding legends.

Reply: We have now updated the Methods to indicate that multi-group comparisons were run in Qlucore Omics Explorer and correction for multiple tests was achieved with the use of q-value cut-off of 0.05 (based on the FDR), which is integral to the software. For cellular assays, each group was compared to the control by unpaired t-test and not multiple comparisons were carried out. To avoid duplication, the different levels of statistical significance denoted by the asterisks were collectedly detailed in the Methods section, under Statistical Analyses, instead of the individual legends.

Comment 3) In Figure 5, panel C, transfection efficiency was estimated by cotransfection of GFP-expressing plasmid, and thus expression levels of Emv2env were not directly measured. The labeling of Emv2env neg, int and high are misleading, unless the authors directly tested the envelope expression levels with a specific antibody.

Reply: We apologise for not making this clearer in the original manuscript. Emv2 env and GFP are in the same viral genome, linked by an internal ribosome entry site (IRES), where a single mRNA produces both proteins at equal ratios. We did not use co-transfection with a GFP expressing plasmid. This is now clarified in the legend.

Editorial issues:
a) In the last line on page 2, "envelops glycoproteins" must be amended.
b) In Figure 5 legend for panel C, "a GFP only carrying virus" must be rephrased.

Reply: These corrections have now been made.

Reviewer #3:

Comment 1. What is the rationale for overexpressing Emv2 envelope in Jurkat cells instead of Xmv45 envelope?

Reply: We aimed to set up clean system where the envelope is not triggered by its cognate receptor expressed in the same cells. The Xmv45 receptor XPR1 is expressed in human cells, whereas the Emv2 receptor mCAT1 is not.

Comment 2. On page 12, the authors state, "RNA-seq analysis over the course of stimulation revealed upregulation of EGR1 and PTCRA and downregulation of RAG1, RAG2, and DLX3 in Jurkat.Emv2env cells stimulated with 83A25 or CD3/CD28, but not in unstimulated cells." However, the data do not show elevated PTCRA in either 83A25 or CD3/CD28 stimulated cells, instead it shows no difference at 3hrs and reduced expression at 18hrs for 83A25-treated cells and at both time points for CD3/CD28-stimulated cells. Similarly, although DLX3 is reduced in transduced cells in the absence of stimulation, it is induced upon both 83A25 and CD3/CD28 stimulation.

Reply: We thank the Reviewer for spotting this error. PTCRA (encoding CD45) and DLX3 had been accidentally swapped in the text (the figure is correct). PTCRA is downregulated and DLX3 is upregulated.

Comment 3. In the discussion, the authors compare their study to antibody-induced internalization of the HIV-envelope. However, the referenced HIV study by Anand et al. (JVI 2019) is based on antibody-dependent cellular cytotoxicity, which is a FcR-dependent process and is therefore not comparable to the existing study given the Fc-independent process described in this manuscript.

Reply: We would respectfully point out that Anand et al., demonstrate internalisation of HIV envelope-antibody complexes in CD4 T cells, analogous to the experiments we describe in this manuscript. This phenomenon was observed simply by incubation of HIV-infected primary CD4 T cells with anti-envelope antibodies and was not FcR dependent. In separate experiments, the authors further showed that this antibody-induced internalisation rendered the cells resistant to antibody-dependent cellular cytotoxicity by autologous PBMCs, which is FcR dependent, but not related to the current study.
